# Isotype-Specific Fc Effector Functions Enhance Antibody-Mediated Rift Valley Fever Virus Protection *In Vivo*

**DOI:** 10.1128/mSphere.00556-21

**Published:** 2021-09-08

**Authors:** Haley N. Cartwright, Dominique J. Barbeau, Anita K. McElroy

**Affiliations:** a University of Pittsburgh, School of Medicine, Department of Pediatrics, Division of Pediatric Infectious Disease, and Center for Vaccine Research, Pittsburgh, Pennsylvania, USA; University of Kentucky College of Medicine

**Keywords:** Fc effector function, IgG1, IgG2a, RVFV, Rift Valley fever virus, MAbs, monoclonal antibodies, protection

## Abstract

Discovered in 1931, Rift Valley fever virus (RVFV) is an arbovirus that causes disease in humans and livestock. In humans, disease ranges from a self-limiting febrile illness to a more severe hepatitis or encephalitis. There are currently no licensed human therapeutics for RVFV disease. Given the recent advances in the use of monoclonal antibodies (MAbs) for treating infectious disease, a panel of anti-RVFV Gn glycoprotein MAbs was developed and characterized. RVFV MAbs spanned a range of neutralizing abilities and mapped to distinct epitopes along Gn. The contribution of Fc effector functions in providing MAb-mediated protection from RVFV was assessed. IgG2a version MAbs had increased capacity to induce effector functions and conferred better protection from RVFV challenge in a lethal mouse model than IgG1 version MAbs. Overall, this study shows that Fc-mediated functions are a critical component of humoral protection from RVFV.

**IMPORTANCE** Rift Valley fever virus (RVFV) is a mosquito-borne virus found throughout Africa and into the Middle East. It has a substantial disease burden; in areas of endemicity, up to 60% of adults are seropositive. With a case fatality rate of up to 3% and the ability to cause hemorrhagic fever and encephalitis, RVFV poses a serious threat to human health. Despite the known human disease burden and the fact that it is a NIAID category A priority pathogen and a WHO priority disease for research and development, there are no vaccines or therapeutics available for RVF. In this study, we developed and characterized a panel of monoclonal antibodies against the RVFV surface glycoprotein, Gn. We then demonstrated therapeutic efficacy in the prevention of RVF *in vivo* in an otherwise lethal mouse model. Finally, we revealed a role for Fc-mediated function in augmenting the protection provided by these antibodies.

## INTRODUCTION

Rift Valley fever virus (RVFV) is a zoonotic arbovirus of the family *Phenuviridae* first identified in 1931 in Kenya (1). RVFV is endemic throughout Africa and the Arabian Peninsula (2), with recent outbreaks across many countries since 2018 (3–6). There is significant risk of spread due to widespread competent mosquito vectors (7–9). Given its potential to cause a public health emergency as well as the absence of human therapeutics or vaccines, WHO has listed Rift Valley fever (RVF) as a priority disease for research and development (10). RVF displays a variety of clinical manifestations, ranging from acute flu-like illness to severe and sometimes lethal hemorrhagic disease or encephalitis (11). Approximately 4,500 cases of severe RVF disease were reported to WHO between 2000 and 2016 (12), although this greatly underestimates the true burden of disease. Serosurveys have revealed widespread seropositivity in humans and animals across Africa (13–17).

Monoclonal antibodies (MAbs) have already shown efficacy in the treatment of multiple infectious diseases, with many in clinical development (18). To date, RVFV MAb research has focused on the development and evaluation of neutralizing MAbs. Neutralization is mediated by the Fab region, which directly contacts a viral surface glycoprotein, blocking entry into host cells. Rabbit, human, monkey, and mouse MAbs directed against the two RVFV glycoproteins—Gn and Gc—have been recently developed and demonstrated protective efficacy in mice (19–23). Gn- and Gc-neutralizing MAbs have demonstrated protection *in vivo* by blocking attachment, entry, or fusion of RVFV (19–21, 23).

In addition to neutralization, antibodies (Abs) provide protection through a variety of mechanisms via their ability to interact with Fc gamma receptors (FcγRs) on innate immune cells. Abs bind FcγRs through their Fc domain to mediate functions, including antibody-dependent cellular cytotoxicity (ADCC), antibody-dependent cellular phagocytosis (ADCP), antibody-dependent neutrophil phagocytosis (ADNP), and complement-dependent cytotoxicity (CDC) (24, 25). The essential role of Fc-mediated immune effector functions in providing protection from viral disease has been reported for Ebola virus, human immunodeficiency virus, influenza A virus, and chikungunya virus (26–31). This suggests the potential for Fc effector functions to be an essential component of MAb-mediated protection from RVFV, a role that has yet to be investigated.

We report the development of a panel of six mouse MAbs against the RVFV Gn glycoprotein. To investigate the contribution of Fc effector functions in antibody-mediated RVFV protection, MAbs were subclass switched to produce IgG1 and IgG2a versions. IgG1 subclass MAbs provided incomplete protection from RVFV disease *in vivo*. However, administration of IgG2a subclass MAbs increased protection to 100% for the three most promising candidates. These results indicate that Fc-effector mechanisms are key components of humoral protection from RVF.

## RESULTS

### Generation and characterization of anti-Gn RVFV MAbs.

A panel of eight RVFV Gn-specific mouse MAbs were generated. These MAbs were selected to span a range of neutralizing and Gn binding abilities based on initial hybridoma cell supernatant enzyme-linked immunosorbent assay (ELISA) and foci reduction neutralization test (FRNT) screening. Antibody variable domain sequencing found MAb-2, -2.2, and -2.3 to be identical, and so six unique MAbs were used throughout the study. Variable domains were cloned into heavy and light chain expression plasmids, and Abs were purified to produce MAb-1, -2, -3, -4, -5, and -6.

**FIG 1:**
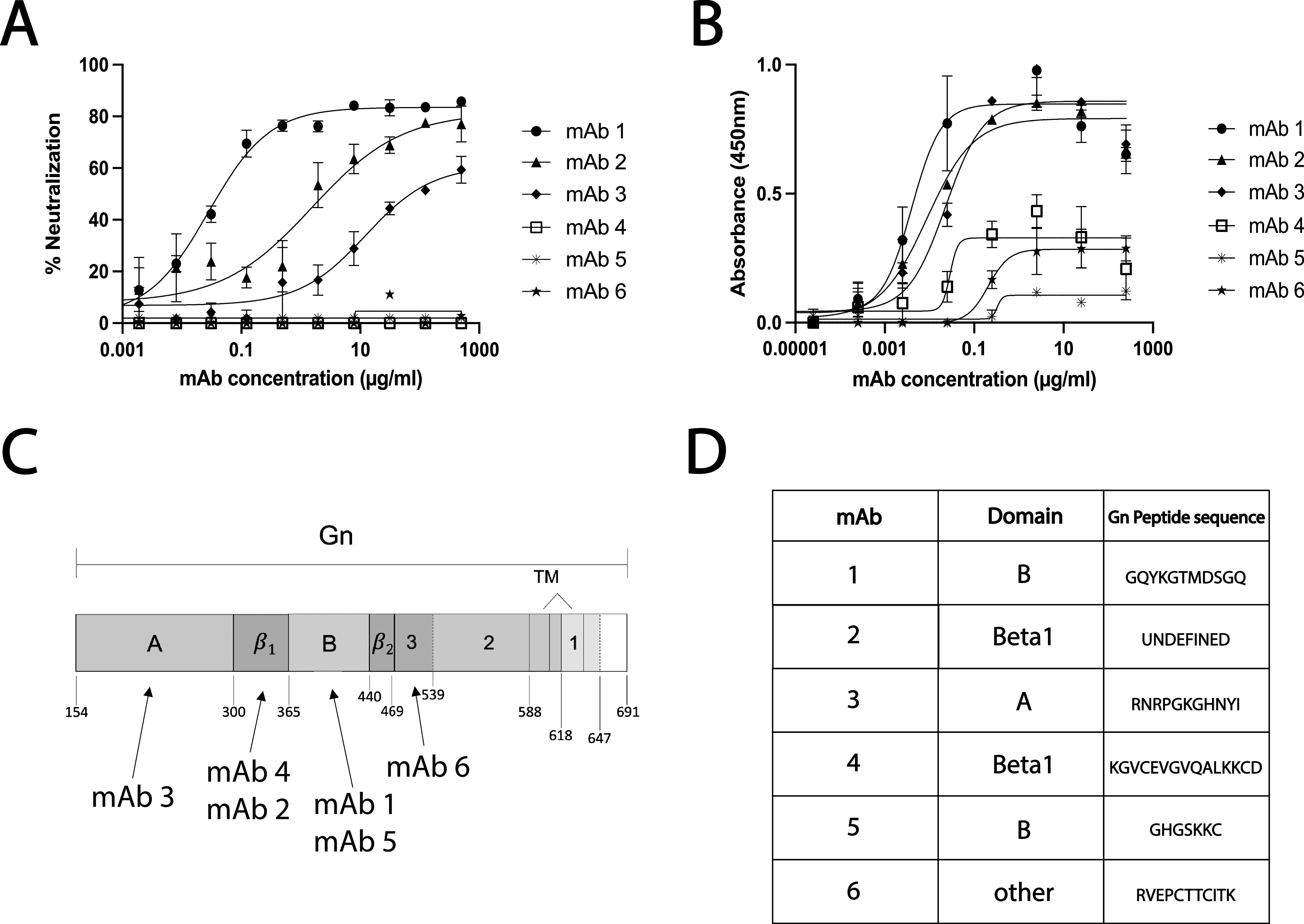
RVFV MAbs display a range of binding and neutralization activities and target domains throughout Gn. (A) The ability of MAbs to neutralize RVFV was assessed by serial dilution of MAbs in an FRNT assay. (B) MAbs were also tested for their ability to bind Gn by RVFV-infected lysate ELISA. Means and standard deviations (SDs) from triplicates are reported for both FRNT and ELISA data. (C) Schematic of the RVFV Gn protein with MAbs mapped to the domain required for binding, as determined through Western blot analysis of truncated Gn constructs. (D) MAbs were mapped to specific epitopes by Gn peptide ELISA.

MAbs displayed a range of RVFV neutralization abilities (Fig. 1A). Three MAbs showed no neutralization ability, even at 500 μg/ml. The half maximal inhibitory concentration (IC_50_) was calculated for each of the three neutralizing MAbs. MAb-1 was the most potently neutralizing, with an IC_50_ of 28 ng/ml followed by MAb-2 (1,532 ng/ml) and MAb-3 (12,260 ng/ml) (see Table S1 in the supplemental material).

10.1128/mSphere.00556-21.1 TABLE S1 Characterization of anti-Gn MAb neutralization and binding. The IC_50_ (half maximal inhibitory concentration) for neutralization and EC_50_ (the effective concentration for 50% binding) are show for each monoclonal antibody. Download Table S1, DOCX file, 0.01 MB. Copyright © 2021 Cartwright et al. 2021 Cartwright et al. https://creativecommons.org/licenses/by/4.0/ This content is distributed under the terms of the Creative Commons Attribution 4.0 International license.

In an RVFV lysate ELISA, all six MAbs were able to bind Gn with varied affinities (Fig. 1B). The lower maximal binding values for MAb-4, -5, and -6 suggest that fewer of these MAbs were able to bind RVFV at saturation than MAb-1, -2, and -3. Fifty percent effective concentration (EC_50_) values ranged from 3.97 to 327.8 ng/ml with MAb-1, -2, and -3 having the lowest values (Table S1).

### Domain and epitope mapping of anti-Gn RVFV MAbs.

Gn truncations were made based on three previously identified structural domains of Gn: A (amino acids [aa] 154 to 300), B (aa 366 to 440), and beta (beta_1_ aa 301 to 365 and beta_2_ 441 to 469) (Fig. 1C) (32, 33). Truncations were also made outside these three domains to split up Gn between the beta_2_ domain and the transmembrane (TM) domain (denoted 1, 2, and 3) (see Fig. S1A). Using these seven Gn truncations (Fig. S1A), the domain required for binding of each MAb (Fig. S1B) was identified. MAbs mapped to different domains along Gn, with MAb-6 binding closest to the TM, outside the previously defined A, B, and beta domains (Fig. 1C; Fig. S1B). Interestingly, the highest neutralizers (MAb-1, -2, and -3) bound different domains of Gn. All MAbs were found to recognize denatured forms of Gn, suggesting linear epitopes.

10.1128/mSphere.00556-21.2 FIG S1 RVFV MAbs target various domains along the length of Gn. (A) Schematic of the RVFV Gn protein with representations of the truncated versions of Gn proteins that were generated for domain mapping. (B) Required domains for binding were mapped by Western Blot analysis. Download FIG S1, PDF file, 0.2 MB. Copyright © 2021 Cartwright et al. 2021 Cartwright et al. https://creativecommons.org/licenses/by/4.0/ This content is distributed under the terms of the Creative Commons Attribution 4.0 International license.

Peptide ELISA was performed to map the epitope recognized by each MAb. All MAbs strongly bound at least one peptide except for MAb-2 (Fig. 1D). Some MAbs bound multiple adjacent and overlapping peptides, which enabled the identification of shorter binding epitopes. All identified binding epitopes were within the domain to which that MAb had previously been mapped by Western blotting. Successful binding to 15-mer peptides by MAbs confirmed that most of these MAbs bound linear epitopes.

### Anti-Gn MAbs increased survival following lethal RVFV challenge.

**FIG 2:**
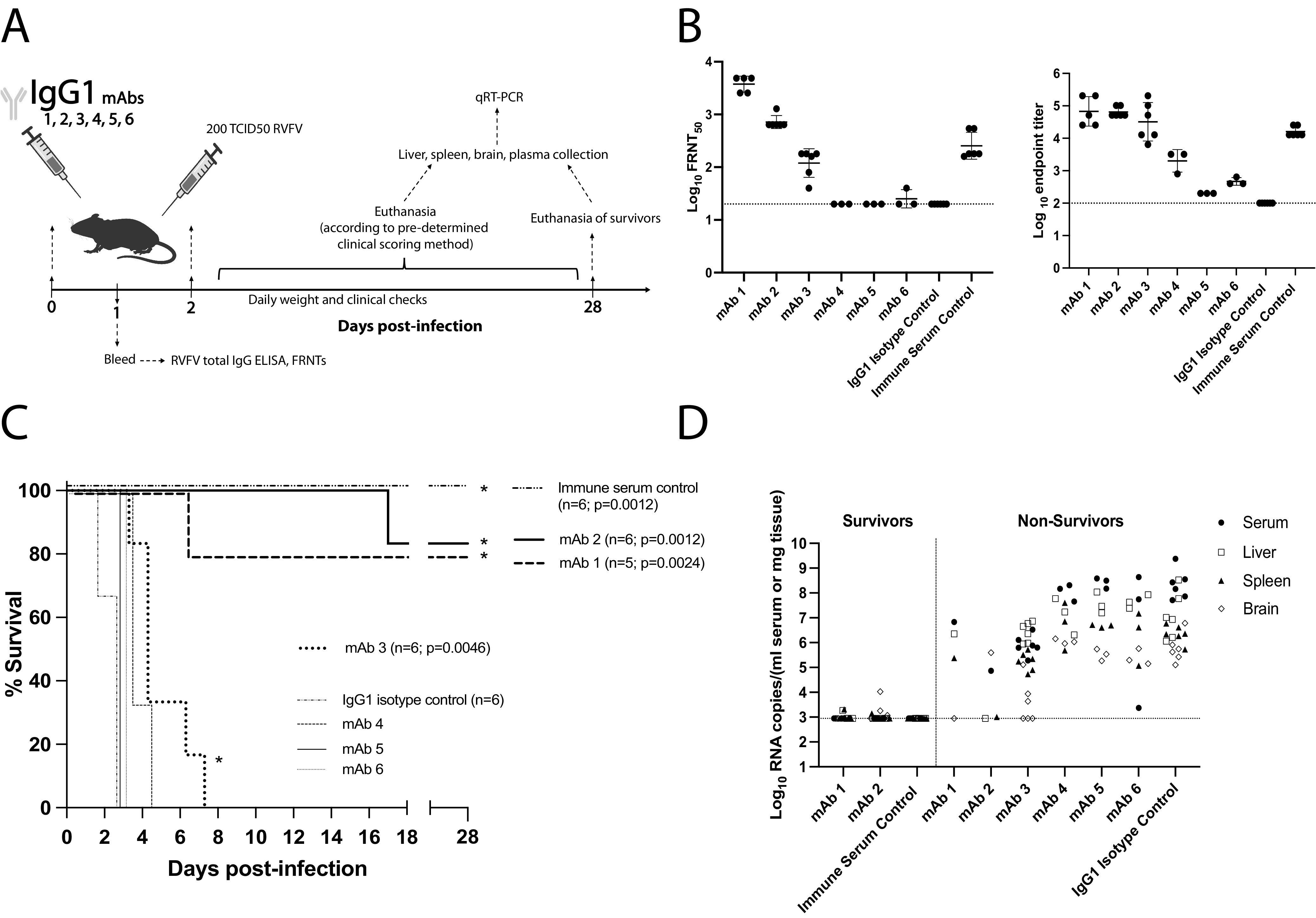
Anti-Gn MAbs increase survival against lethal RVFV challenge. (A) Schematic of the IgG1 MAb-treated mouse survival experiment. (B) FRNT and ELISA of serum collected 24 h after MAb injection. Geometric mean titers are shown with a horizontal line, and error bars represent the geometric SD for each MAb treatment group. LOD of each assay is noted by dotted line. (C) Survival curve of mice following challenge. Positive-control mice injected with RVFV immune serum all survived challenge and exhibited a statistically significant difference in survival compared to that of IgG1 isotype control-treated mice (Mantel-Cox test; *P* = 0.0012). MAb-1 and -2 also showed equally significant differences in survival compared to that of IgG1 isotype control-treated mice, although there was not 100% protection from lethal RVFV challenge (Mantel-Cox test; MAb-1 *P* = 0.0024; MAb-2 *P* = 0.0012). *, significance in survival compared to IgG1 isotype control survival. (D) qRT-PCR based assessment of viral RNA loads in tissues and serum at time of euthanasia. Surviving mice were euthanized 28 days postinfection (dpi). The LOD for this assay is reported as a horizontal dashed line at 887 RNA copies.

C57BL/6 mice are an RVFV lethal challenge model, succumbing to infection within 4 days (34). To determine the protective potential of these anti-Gn MAbs, 400 μg of each IgG1 MAb was administered via intraperitoneal (i.p.) injection 48 h prechallenge with 200 times the 50% tissue culture infective dose (TCID_50_) of wild-type (WT) RVFV (Fig. 2A). Serum FRNT and ELISA at 24 h postinjection (Fig. 2B) confirmed MAb administration in all mice.

IgG1 isotype control-treated mice succumbed to disease within 3 days of challenge, while positive-control mice given RVFV-vaccinated immune serum survived to the end of the experiment (Fig. 2C). MAb-1 and -2 protected mice significantly better than the isotype control, with only one mouse per experimental group succumbing to disease. Although MAb 3-treated mice all succumbed to disease, their survival curve was different from that of the isotype control, with a significantly increased time to death. Mice treated with nonneutralizing MAb-4, -5, or -6 all succumbed to disease with no significant delay in time to death. Weight loss was appreciated in all mice that succumbed to acute hepatic death (see Fig. S2). At the point of euthanasia, tissues and serum were assessed for RVFV RNA loads. Elevated viral RNA throughout the tissues and serum confirmed that mice succumbed due to RVFV infection (Fig. 2D). Notably, although mice treated with MAb-3 all succumbed, decreased levels of viral RNA suggest some level of MAb-mediated viral control (Fig. 2D). In survivor mice, RNA levels were at or near the limit of detection (LOD) by day 28 postchallenge, suggesting overall control of the virus (Fig. 2D).

10.1128/mSphere.00556-21.3 FIG S2 Weight loss of IgG1 and IgG2a MAb-treated mice. Percent change in mouse daily weight from baseline in either IgG1 (A) or IgG2a (B) MAb-treated mice. Weight loss curves represent six female mice for a given MAb treatment group. Download FIG S2, EPS file, 1.5 MB. Copyright © 2021 Cartwright et al. 2021 Cartwright et al. https://creativecommons.org/licenses/by/4.0/ This content is distributed under the terms of the Creative Commons Attribution 4.0 International license.

### Subclass switching of anti-Gn RVFV MAbs.

To test the hypothesis that protection delivered by the three partially protective MAbs could be enhanced by increasing their ability to induce Fc effector functions, the inherent differential abilities of murine IgGs to interact with FcγRs on innate immune cells was utilized. In mice, IgG2a Abs induce high effector function strength by binding the high-affinity activating receptors FcγRIV and FcγRI and the low-affinity receptor FcγRIII. IgG1 Abs signal through FcγRIII but not FcγRIV or FcγRI and are thus classically thought to affect lower levels of Fc-mediated defense (35–38). Therefore, to increase the ability of the RVFV-Gn IgG1 MAbs to induce Fc-mediated immune effector functions, they were subclass switched to IgG2a.

**FIG 3:**
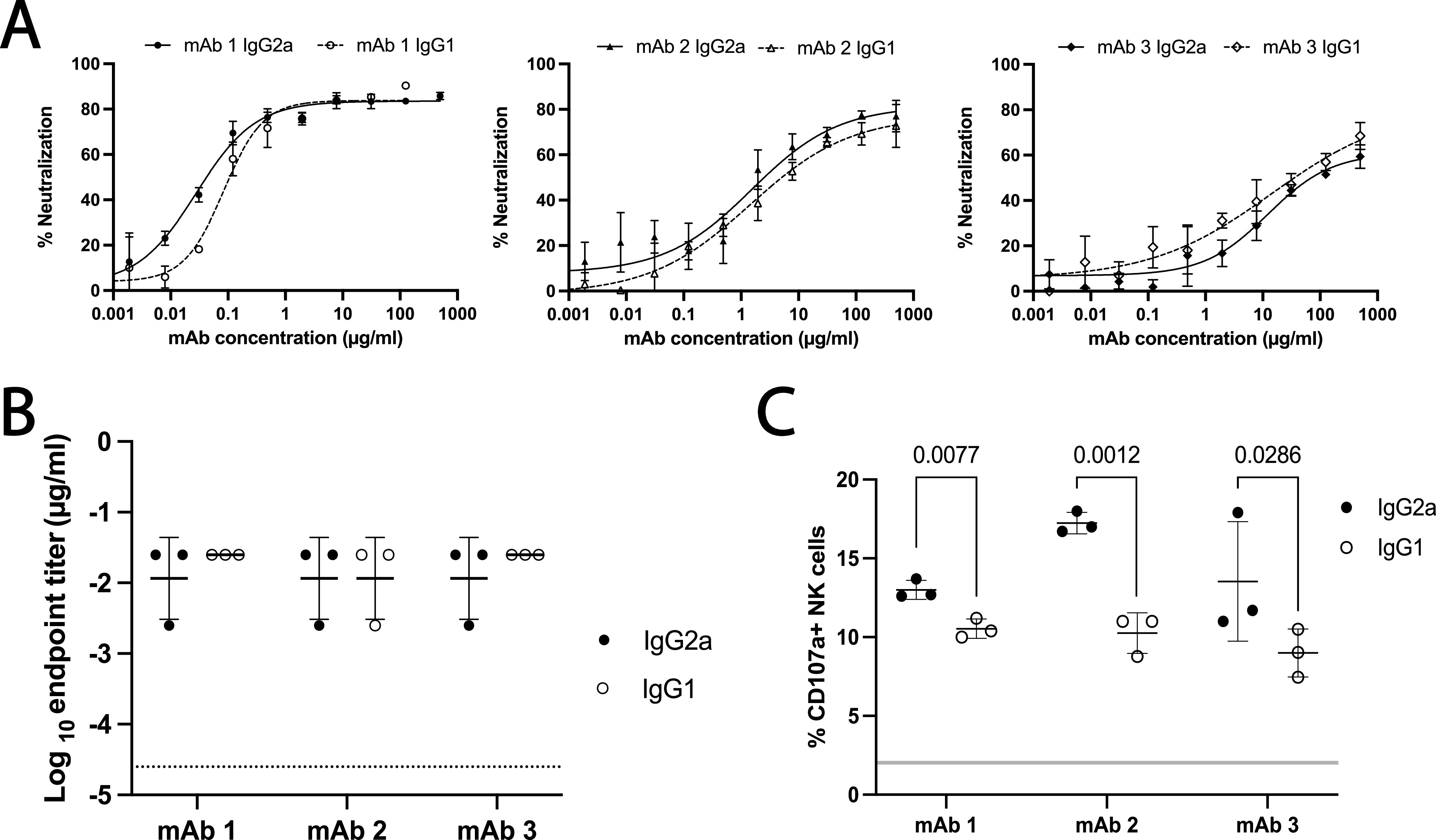
Subclass-switched MAbs display similar binding and neutralization but increased ability to activate effector functions. (A) Subclass-switched MAbs had similar neutralizing ability as assessed by FRNT. Mean and SD in triplicate are reported. (B) Subclass-switched MAbs had similar Gn binding as tested by ELISA of RVFV-infected lysates. Geometric means are shown with a horizontal line, and error bars represent the geometric SD for each MAb. The horizontal dashed line represents the LOD of this assay. (C) Assessment of MAb ADCC by NK cell degranulation assay. Each point represents an independent replicate, with means shown by a horizontal line and SD by the error bars. The gray horizontal line indicates the range of degranulation induced by IgG1 and IgG2a isotype control Abs. Statistical significance in the ability of MAbs to induce degranulation in NK cells was assessed by unpaired Student’s *t* tests. (MAb-1, *P* = 0.0077; MAb-2, *P* = 0.0012; MAb-3, *P* = 0.0286).

Functionality of IgG2a version MAbs that had shown some level of protection *in vivo* was assessed by FRNT and ELISA. Subclass-switched MAbs exhibited an RVFV neutralization capacity similar to that of their IgG1 counterparts (Fig. 3A). Subclass-switched MAbs also bound Gn with affinities resembling those of their IgG1 versions, confirmed by ELISA (Fig. 3B). To confirm that IgG2a version MAbs successfully induced higher effector functions, their ability to induce Gn/MAb interaction-dependent activation of NK cells was assessed by NK cell degranulation (% CD107a^+^ NK cells) (Fig. 3C; see also Fig. S3). The IgG2a version of all three MAbs showed significantly higher NK cell degranulation (Fig. 3C).

10.1128/mSphere.00556-21.4 FIG S3 Identification and characterization of NK cells. Flow cytometry gating strategy used in the identification of NK cells and the quantification of CD107a^+^ NK cells. Doublets and dead cells were excluded from total selected lymphocytes. NK cells were discriminated from any remaining cells as NK1.1^+^, and from this population, the percentage of NK cells positive for CD107a was quantified. A negative-control sample is shown. Download FIG S3, PDF file, 0.1 MB. Copyright © 2021 Cartwright et al. 2021 Cartwright et al. https://creativecommons.org/licenses/by/4.0/ This content is distributed under the terms of the Creative Commons Attribution 4.0 International license.

### Protection *in vivo* was enhanced by Fc effector function.

**FIG 4:**
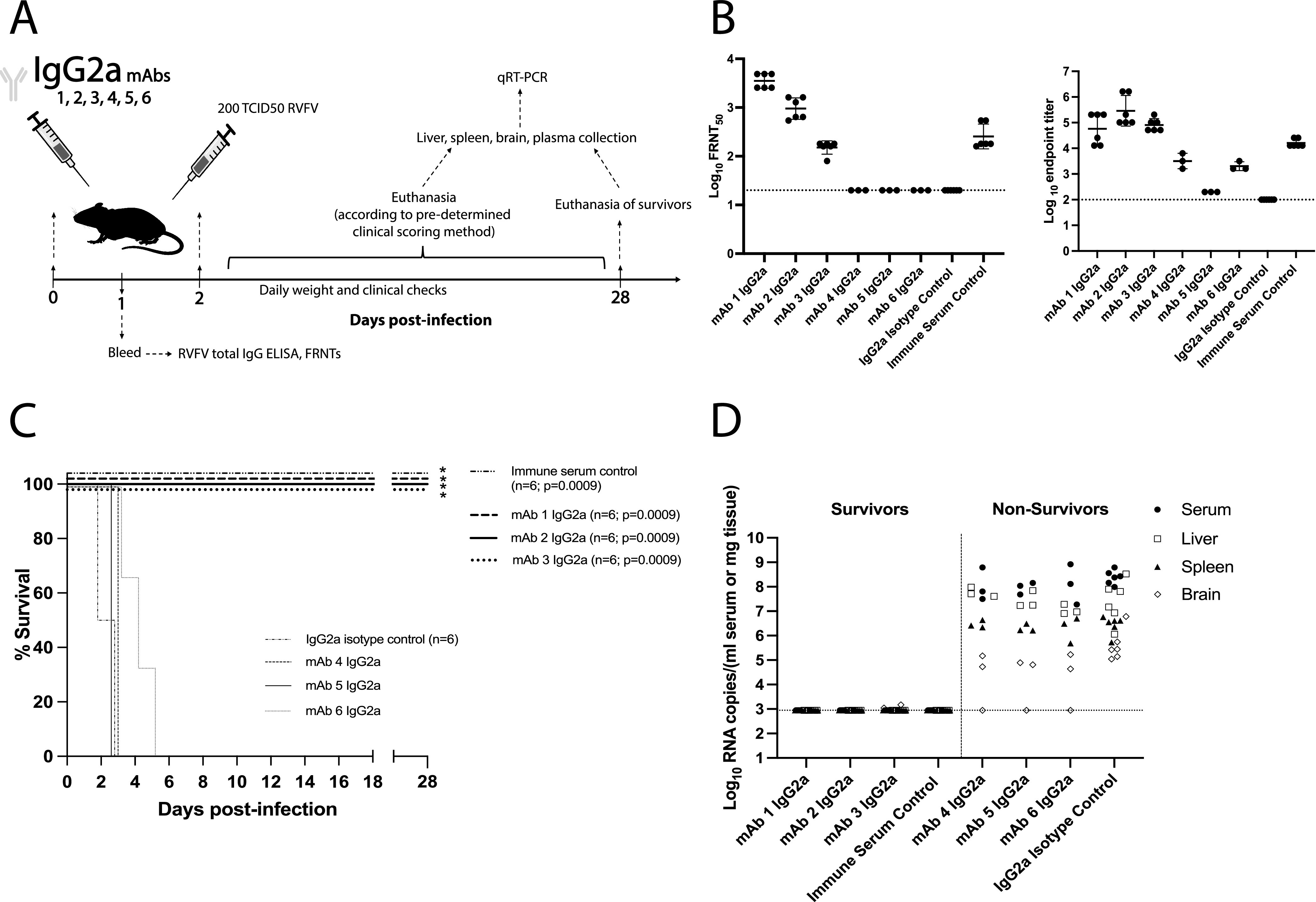
IgG2a MAbs confer increased protection against lethal RVFV challenge. (A) Schematic of the IgG2a MAb-treated mouse survival experiment. (B) FRNT and ELISA of bleed serum taken 24 h after MAb injection. Geometric means are shown with a horizontal line, and error bars represent the geometric SD for each MAb treatment group. LOD of each assay is noted by the dotted line. (C) Survival curve of mice following challenge. Positive-control mice injected with RVFV immune serum all survived challenge and exhibited a statistically significant difference in survival compared to IgG2a isotype control-treated mice (Mantel-Cox test; *P* = 0.0009). The IgG2a version of MAb-1, -2, and -3 all displayed complete protection from lethal challenge, and the survival curves were significant compared to those of IgG2a isotype control-treated mice (Mantel-Cox test, MAb-1, -2, and -3, *P* = 0.0009). *, significance in survival compared to IgG2a isotype control survival. (D) qRT-PCR based assessment of viral RNA loads in tissues and serum at time of euthanasia. Surviving mice were euthanized 28 dpi. The LOD for this assay is reported as a horizontal dashed line at 887 RNA copies.

To test whether Fc effector functions were important for RVFV protection *in vivo*, IgG2a version MAbs were administered to C57BL/6 mice prechallenge as before (Fig. 4A). FRNT and ELISA of mouse bleed serum 24 h postinjection revealed appropriate levels and functionality of the administered IgG2a MAbs (Fig. 4B).

IgG2a isotype control-treated mice succumbed to lethal RVFV challenge similarly to the IgG1 isotype controls, while all positive-control mice survived (Fig. 4C). IgG2a MAb-1, -2, and -3 fully protected mice from lethal challenge, with their survival curves being significant compared to those of IgG2a isotype control-treated mice. All mice administered a nonneutralizing IgG2a (MAb-4, -5, or -6) still succumbed to disease. Viral RNA loads were high in all mice that succumbed and were at or below the LOD in surviving mice (Fig. 4D).

To determine if protection provided by MAb-1, -2, and -3, was sterilizing, results from ELISA and FRNT on terminal survivor mouse serum were compared to bleed antibody titers measured at 24 h prechallenge (see Fig. S4). These data showed an overall increase in total antibody titers and neutralization in the serum of mice treated with MAbs that survived RVFV infection (Fig. S4A). RVFV N protein-specific ELISA was performed on terminal survivor serum to confirm that antibody titer increases were due to *de novo* production of antibody within the mice in response to infection (Fig. S4B). The presence of anti-N antibody titers in all survivor MAb-treated mice, regardless of subclass, confirmed that protection by these anti-Gn MAbs was not sterilizing.

10.1128/mSphere.00556-21.5 FIG S4 Anti-Gn MAbs confer nonsterilizing protection from lethal RVFV challenge regardless of subclass. (A) Comparison of FRNT and ELISA data at 24 h postadministration (prechallenge bleed, black circles) with terminal bleed from all surviving animals (28 days postchallenge, open circles). There are no terminal data for MAb-3 IgG1 due to all mice succumbing to disease before day 28. Geometric mean titers are shown with a horizontal line, and error bars represent the geometric SD for each MAb treatment group either pre- (solid) or postchallenge (dotted). The horizontal dashed lines represent the LOD of these assays. All mice that survived RVFV challenge developed anti-N antibody titers. (B) Geometric means are shown with a horizontal line and error bars represent the geometric SD for each MAb treatment group. The horizontal dashed line represents the LOD of this assay. Download FIG S4, EPS file, 1.6 MB. Copyright © 2021 Cartwright et al. 2021 Cartwright et al. https://creativecommons.org/licenses/by/4.0/ This content is distributed under the terms of the Creative Commons Attribution 4.0 International license.

## DISCUSSION

Previous studies have investigated the importance of neutralizing MAbs in protection from lethal RVFV disease. Neutralizing MAbs raised against Gn or Gc protect by various mechanisms of virus neutralization, including the blocking of attachment, entry, or fusion (19–21, 23). When each MAb described in this study was mapped to its Gn binding domain, all except MAb-2 were found to bind linear epitopes. This overwhelming recognition of linear epitopes was not surprising, as the immunogen was produced in bacteria and may not have had its native confirmation. The MAbs in this study bound to epitopes across the three domains of Gn, with the highest neutralizer (MAb-1) binding a new site of vulnerability in domain B. This domain has been suggested as an immunodominant region of Gn, with others having mapped protective MAbs to this region (19). Other work has identified domain A as a hot spot for binding of highly neutralizing MAbs (20, 23). The lowest of the three neutralizers, MAb-3, bound a new site of vulnerability in domain A distinct from those for previously identified MAbs. These novel protective epitopes point to the probability that the entire outward facing surface of Gn can be targeted by MAbs to elicit protection *in vivo*.

Despite published work regarding RVFV-neutralizing Abs providing protection from disease, the reliance on Fc effector function for delivering said protection was not previously assessed. This study investigated the role of Fc effector functions in MAb-mediated RVFV protection using the divergent effector function strengths of IgG subclass MAbs. A panel of MAbs was developed against the RVFV Gn glycoprotein, and each MAb was cloned to be both IgG1 and IgG2a subclass. When administered to mice prechallenge, protection from RVFV disease was enhanced by IgG2a subclass MAbs.

Indeed, protection from lethal RVFV challenge was dependent on the functions provided by the IgG2a Fc domain, as MAb-1, -2, and -3 only afforded complete protection when administered as the IgG2a subclass. A significant difference in survival was seen between MAb-3 IgG2a- and IgG1-treated groups (Mantel-Cox test *P* = 0.0005) (Fig. 2C and 4C). MAb-1 and -2 did not show a statistically significant difference in survival between subclasses, but IgG2a-administered mice were provided a clear survival advantage. Lack of statistical significance between subclasses for MAb-1 and -2 was likely due to relatively small sample sizes. MAb-3 provided the greatest increase in protection when the subclass was switched to IgG2a, with survival outcomes changing from 0% to 100%. This large increase was ostensibly due to MAb-3 being the lowest level neutralizer, as all three partially protective IgG1 MAbs elicited similar levels of NK cell degranulation. This suggests that humoral protection from RVFV likely requires a level of contribution from nonneutralizing mechanisms that changes depending on the neutralizing strength of the response.

The protective capacity of each MAb was unequally enhanced, however, when given as the IgG2a version. MAb-4, -5, and -6 failed to protect mice from death regardless of antibody subclass. This difference in protection between MAbs could be due to the lower binding affinity of MAb-4, -5, and -6. Binding with high enough affinity to the antigen to induce immune complex formation is known to be crucial for the activation of Fc effector functions (39, 40). The inability of MAb-4, -5, and -6 to protect mice could alternatively be due to their nonneutralizing status. Optimal protection from RVFV may require both neutralization and Fc-dependent effector functions, as seen for other viruses (26, 28, 41). Future work is required to test whether nonneutralizing MAbs with strong Gn binding affinity can protect from RVFV *in vivo*.

In addition to increasing survival, IgG2a MAbs also seemed to control viral infection more efficiently. Viral RNA was detected at day 28 at low levels in IgG1 MAb-1 and -2 surviving mice. Contrastingly, RNA levels were below the LOD for survivor mice treated with the IgG2a version. Decreased viral titers were also seen in the brains of mice treated with the nonprotective, nonneutralizing IgG2a version of MAbs-4, -5, and -6. This might suggest increased control of viral replication and/or spread to the brain by IgG2a MAbs. This accelerated viral clearance by IgG2a MAbs may mitigate the development potential of late-onset encephalitis. Individuals presenting for care with RVF are typically well into the disease course. Therefore, it will be important to determine if MAb therapy can prevent progression to late-onset encephalitis and the role of Fc effector functions therein, as this is where MAbs have the most human promise. Recent work in humans found that the antibody response to naturally acquired infection preferentially targets Gn, with neutralizing anti-Gn IgG responses lasting decades (42). Future investigation into the contribution of Fc effector functions in the human humoral response could further increase understanding of how antibody-mediated immunity protects against RVFV disease.

## MATERIALS AND METHODS

### Ethics statement and biosafety information.

Animal research was approved by University of Pittsburgh IACUC (protocol 19044158). All experiments with the wild-type (WT) RVFV ZH501 strain were performed in the University of Pittsburgh regional biocontainment biosafety level 3 laboratory.

### Virus generation.

WT RVFV, DelNSs RVFV, and DelNSs/DelNSm RVFV were generated using reverse genetics based on the ZH501 strain background (43–45). Virus stocks were grown to passage 2 and fully sequence confirmed using next-generation sequencing prior to use. Viral stock titers were determined by 50% tissue culture infective dose (TCID_50_) assay as described previously (34, 46).

### MAb generation.

Custom mouse hybridomas were generated commercially by GenScript. Briefly, 5 BALB/c and 5 C57BL/6 mice were immunized 3 times with bacterially produced RVFV Gn protein. Splenocytes from three mice with the highest RVFV Gn-specific ELISA titers were fused to SP/0 myeloma cells to generate hybridomas. Hybridoma supernatants were screened for antibody reactivity via ELISA and FRNT. Six hybridoma clones, naturally derived as 1 IgG2a and 5 IgG1, spanning a range of neutralization and binding abilities were selected for antibody production and purification. Antibody variable domains were sequenced and cloned into heavy and light chain expression plasmids pFUSEss-CHIg-mG1/pFUSEss-CHIg-mG2a and pFUSE2ss-CLIg-mk (InvivoGen). Heavy and light chain plasmids were cotransfected into FreeStyle 293-F suspension cells using 293fectin. Cells were cultured in FreeStyle 293 expression medium (Thermo Fisher) for 4 days. Secreted Abs were purified by protein G affinity chromatography from cell supernatants (Thermo Fisher).

### MAb domain mapping.

Gn truncations (see Fig. S1 in the supplemental material) were cloned into pcDNA3.1 under the cytomegalovirus (CMV) promoter and then transfected into Vero-E6 cells. Lysates were harvested at 48 h in 50 mM dithiothreitol LDS buffer (Thermo Fisher). Samples were heated at 70°C and then loaded into 4 to 12% bis-Tris gels (Thermo Fisher). Proteins were transferred to nitrocellulose membranes using a Mini Blot module wet transfer system (Thermo Fisher). Membranes were blocked in 5% nonfat dry milk (NFDM) in phosphate-buffered saline with 0.1% Tween 20 (PBST) for 1 h and then probed with each anti-Gn MAb, diluted 1:1,000. Bound MAbs were detected using anti-mouse IgG conjugated to horseradish peroxidase (HRP) (Jackson ImmunoResearch), diluted 1:15,000. Membranes were incubated in SuperSignal West Dura extended duration substrate (Thermo Fisher) for 2 min before exposure to CL-XPosure Film (Thermo Fisher) and developed using an SRX-101A film processor (Konica Minolta).

### MAb epitope mapping.

Overlapping peptides with >70% purity (15-mers with 11-aa overlaps) were generated to span RVFV Gn (GenScript). MaxiSorp plates (Thermo Fisher) were coated one peptide per well with 1 μM each Gn peptide. Plates were incubated at 4°C overnight and then blocked in 1% bovine serum albumin (BSA) in PBST (0.01%) at 37°C for 1 h. After washing in 0.05% PBST, 0.25 μg/ml of each MAb was incubated on blocked plates at 37°C for 2 h. Plates were washed and then incubated for 1 h at 37°C in anti-mouse IgG-HRP (Jackson ImmunoResearch) diluted 1:5,000. Plates were developed in tetramethylbenzidine (TMB) and stopped with TMB stop solution (Seracare). Plates were read at 450 nm, and wells were considered positive if the raw optical density (OD) was >1.

### Mouse study design.

Six- to 8-week-old female C57BL/6J (stock number 000664) mice were purchased from Jackson Laboratories. Mice were housed in HEPA filtration racks with *ad libitum* access to food and water. Mice were administered 400 μg of MAb or 200 μl of RVFV immune serum (derived from mice vaccinated with either DelNSs or DelNSs/DelNSm RVFV) by intraperitoneal (i.p.) injection 48 h prechallenge. Isotype control MAbs were *InVivo*Plus mouse isotype control, unknown specificity (IgG1 clone MOPC-21, IgG2a clone C1.18.4; BioXcell). Twenty-four hours post-MAb administration, serum was obtained via lateral saphenous bleed. Following infection by footpad injection with 200 TCID_50_ recombinant WT RVFV, mice were evaluated for clinical signs of disease and weighed daily as previously described (34).

### Quantitative RT-PCR.

RNA was extracted from tissue samples with TRIzol reagent, and quantitative reverse transcription-PCR (qRT-PCR) targeting the L segment of RVFV (47) was performed (34). RNA copies for each unknown sample were determined by comparison to a standard L RNA curve and normalized by tissue weight or serum volume. The assay’s lowest limit of detection (LOD) is reported on all graphs at 887 RNA copies. The LOD was calculated as the highest threshold cycle (*C_T_*) value detected in the standard curve multiplied by 50, to account for dilutions, and divided by the average sampled tissue weights.

### Enzyme-linked immunosorbent assay.

ELISAs were performed as described previously (34) using plates coated with RVFV-infected Vero-E6 cell lysate or with 200 ng/well of purified RVFV N protein (custom; GenScript). Endpoint ELISA titers for lysate and anti-N protein ELISAs were defined as the highest dilution of serum that resulted in an OD value at least two standard deviations above the average obtained from all negative mouse serum control wells. EC_50_s were calculated by fitting raw OD ELISA data, in triplicates, with a nonlinear least-squares regression best fit curve.

### Foci reduction neutralization test.

Mouse serum or MAb was serially diluted, in duplicates, and incubated with 200 foci-forming units of DelNSs/DelNSm RVFV as described previously (48). Foci were detected using Moss TMB-H peroxidase substrate (MossBio) and counted using an immunospot reader (CTL). Percent neutralization was calculated by comparing sample wells to wells containing virus but no serum/antibody. The concentration of MAb or dilution of serum at which 50% of foci were neutralized is reported as FRNT_50_.

### Antibody-dependent cellular cytotoxicity.

Three micrograms per milliliter RVFV Gn protein (custom; GenScript)-coated MaxiSorp plates (Thermo Fisher) were blocked with 5% BSA in PBST (0.01%) for 1 h at 37°C. MAbs were added to wells at 5 μg/ml and incubated for 2 h at 37°C. NK cells were isolated by negative selection from C57BL/6 mouse spleens using EasySep mouse NK cell isolation kit (StemCell Technologies). Purified NK cells were added at 2 × 10^5^ cells/well in the presence of brefeldin A (Sigma-Aldrich), GolgiStop (BD), and anti-CD107a conjugated to phycoerythrin (PE) (BioLegend clone 1D4B) to wells already containing Gn/MAb. NK cells were incubated for 5 h at 37°C. Cells were then washed and stained with near-infrared (IR) fluorescent reactive dye (Thermo Fisher). Cells were stained for cell surface markers CD3 allophycocyanin (APC)-Cy7 (BioLegend clone 17A2), CD11b fluorescein isothiocyanate (FITC) (BioLegend clone M1/70), and NK1.1 APC (BioLegend clone PK136). The purity of NK cells was confirmed by CD3 APC (BioLegend clone 17A2), CD19 BV421 (BioLegend clone 6D5), NKp46 PE-Cy7 (Biolegend clone 29A1.4), and CD14 APC-Cy7 (BioLegend clone Sa14-2) staining. All cells were fixed in BD Cytofix/Cytoperm and then analyzed by flow cytometry on a BD LSRFortessa flow cytometer. All flow cytometric data were analyzed using FlowJo 10.7.1.

### Statistics.

Data were entered into GraphPad Prism 9 for statistical analysis and graphing. Survival curves were compared using a log rank (Mantel-Cox) test. Unpaired Student’s *t* tests were used to compare ADCC data between MAb subclasses. qRT-PCR data were analyzed in Excel. Specific statistical tests for each data set are indicated in the figure legends.

### Data availability.

The NCBI accession numbers for the antibodies described in this paper are as follows. Each of the six MAbs has two accession numbers, one corresponds to the antibody heavy chain, the other to the light chain. MAb name in this paper (BankIt name on NCBI), NCBI accession number: MAb-1 (23A8-1HeavyChain), MZ998921; MAb-1 (23A8-1LightChain), MZ998922; MAb-2 (23B5-1HeavyChain), MZ998923; MAb-2 (23B5-1LightChain), MZ998924; MAb-3 (32A8-1HeavyChain), MZ998925; MAb-3 (32A8-1LightChain), MZ998926; Mab-4 (30D1-1HeavyChain), MZ998927; MAb-4 (30D1-1LightChain), MZ998928; MAb-5 (21E9-1HeavyChain), MZ998929; MAb-5 (21E9-1LightChain), MZ998930; MAb-6 (33A11-1HeavyChain), MZ998931; MAb-6 (33A11-1LightChain), MZ998932.
