# Supplementary material for: Isotype-Specific Fc Effector Functions Enhance Antibody-Mediated Rift Valley Fever Virus Protection In Vivo
Source: mSphere. 2021 Sep 8;6(5):e00556-21. doi: 10.1128/mSphere.00556-21 (PMC8550229; doi:10.1128/mSphere.00556-21)
Supplement: TABLE S1 [file msphere.00556-21-st001.docx]

**Supplemental Table** Characterization of anti-Gn mAb neutralization and binding

| **mAb** | **IC_50_* for neutralizing RVFV**  **(ng/ml)** | **EC_50_** for binding Gn**  **(ng/ml)** |
| --- | --- | --- |
| 1 | 28.06 | 3.97 |
| 2 | 1,532 | 8.77 |
| 3 | 12,260 | 23.04 |
| 4 | N/A | 28.73 |
| 5 | N/A | 327.8 |
| 6 | N/A | 206.8 |

| *The half maximal inhibitory concentration (IC50)  **The effective concentration of 50% binding (EC50)  mAb, monoclonal antibody; RVFV, Rift Valley Fever virus. |
| --- |
