# Supplementary figures and images for: Isotype-Specific Fc Effector Functions Enhance Antibody-Mediated Rift Valley Fever Virus Protection In Vivo
Source: mSphere. 2021 Sep 8;6(5):e00556-21. doi: 10.1128/mSphere.00556-21 (PMC8550229; doi:10.1128/mSphere.00556-21)

# A

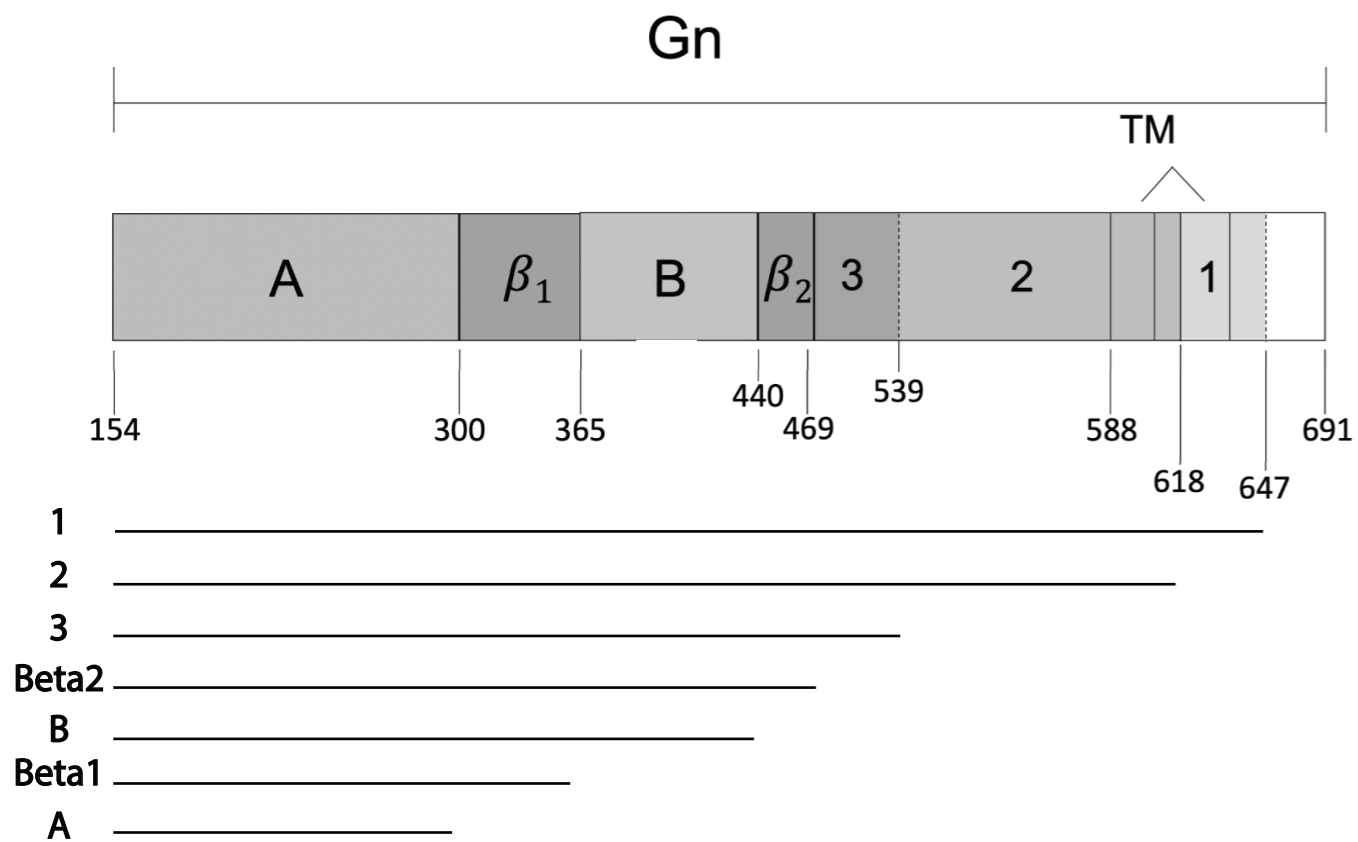

# B

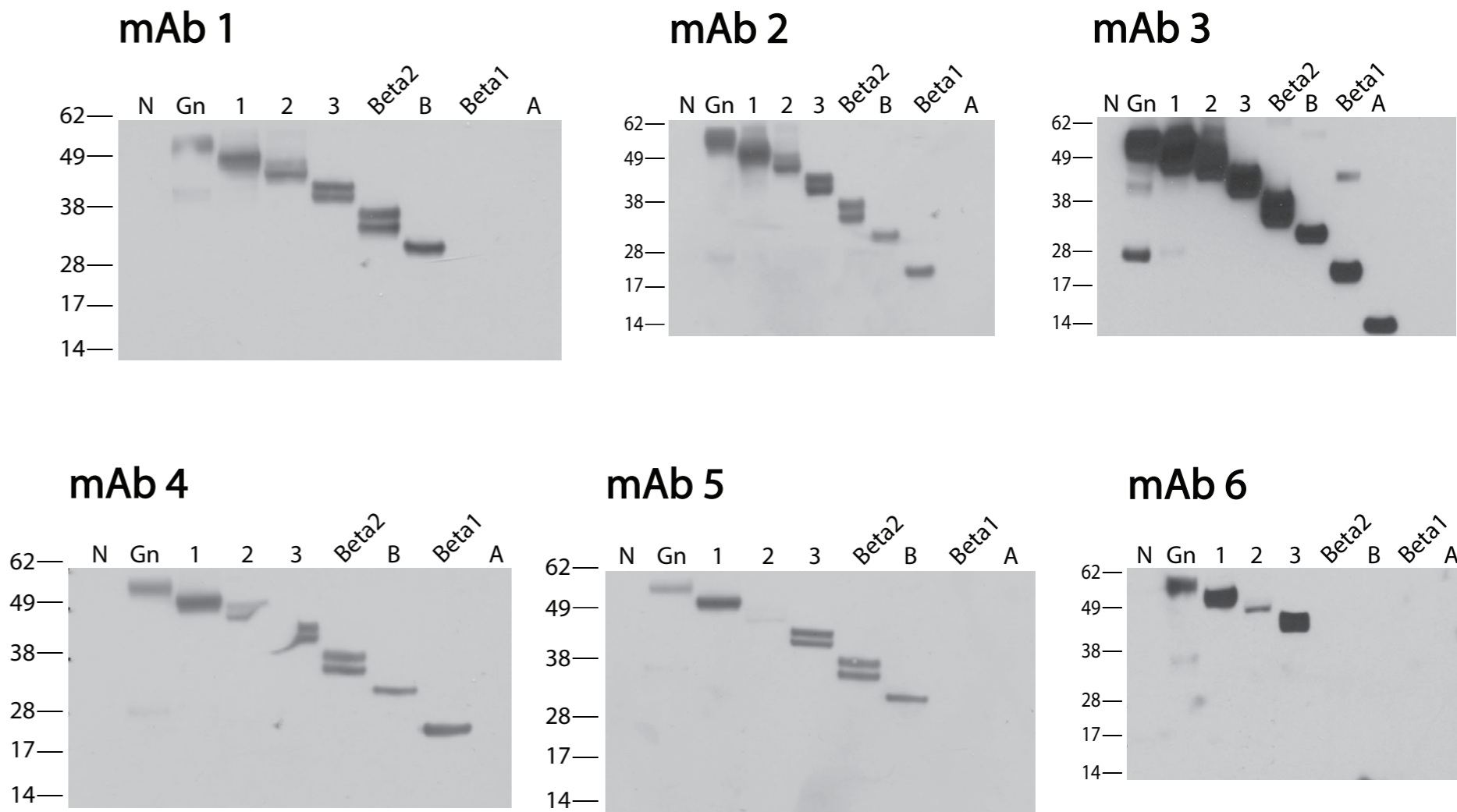

Supplement: FIG S1 [file msphere.00556-21-sf001.pdf]
