# Supplementary material for: Isotype-Specific Fc Effector Functions Enhance Antibody-Mediated Rift Valley Fever Virus Protection In Vivo
Source: mSphere. 2021 Sep 8;6(5):e00556-21. doi: 10.1128/mSphere.00556-21 (PMC8550229; doi:10.1128/mSphere.00556-21)
Supplement: FIG S3 [file msphere.00556-21-sf003.pdf]

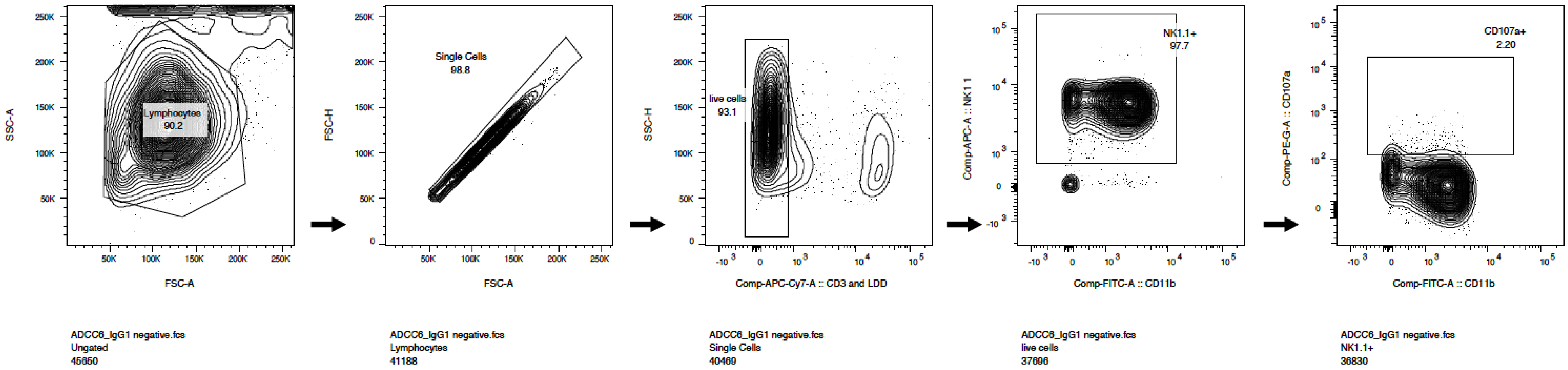

Lymphocytes
90.2
SSC-A
FSC-A
ADCC6_IgG1 negative.fcs
Ungated
45650
Single Cells
98.8
FSC-H
FSC-A
ADCC6_IgG1 negative.fcs
Lymphocytes
41188
live cells
93.1
SSC-H
Comp-APC-Cy7-A :: CD3 and LDD
ADCC6_IgG1 negative.fcs
Single Cells
40469
NK1.1+
97.7
Comp-APC-A :: NK1.1
Comp-FITC-A :: CD11b
ADCC6_IgG1 negative.fcs
live cells
37696
CD107a+
2.20
Comp-PE-G-A :: CD107a
Comp-FITC-A :: CD11b
ADCC6_IgG1 negative.fcs
NK1.1+
36830
